# Prevalence and predictors of post-abortion modern contraceptive utilization among reproductive age women in Ethiopia

**DOI:** 10.1038/s41598-023-42911-7

**Published:** 2023-09-23

**Authors:** Tadele Biresaw Belachew, Wubshet Debebe Negash, Desale Bihonegn Asmamaw, Samrawit Mihret Fetene, Banchlay Addis, Tsegaw Amare Baykeda, Atitegeb Abera Kidie, Abel Endawkie, Alebachew Ferede Zegeye, Tadesse Tarik Tamir, Elsa Awoke Fentie, Sisay Maru Wubante

**Affiliations:** 1 Department of Health Systems and Policy, Institute of Public Health, College of Medicine and Health Sciences, University of Gondar, P. O. Box: 196, Gondar, Ethiopia; 2 Department of Reproductive Health, Institute of Public Health, College of Medicine and Health Sciences, University of Gondar, Gondar, Ethiopia; 3 School of Public Health, The University of Queensland, Brisbane, Australia; 4 School of Public Health, College of Health Science, Woldia University, Woldia, Ethiopia; 5 Department of Epidemiology and Biostatistics, School of Public Health, College of Medicine and Health Science, Wollo University, Wollo, Ethiopia; 6 Department of Medical Nursing, School of Nursing, College of Medicine and Health Sciences, University of Gondar, Gondar, Ethiopia; 7 Department of Pediatric and Child Health Nursing, School of Nursing, College of Medicine and Health Sciences, University of Gondar, Gondar, Ethiopia; 8 Department of Health Informatics, Institute of Public Health, College of Medicine and Health Sciences, University of Gondar, Gondar, Ethiopia

**Keywords:** Health care, Health occupations

## Abstract

The development of a post-abortion family plan is an integral part of comprehensive abortion care. In spite of this, it received insufficient attention as a means of breaking the cycle of repeated abortions, unintended pregnancies, and maternal deaths resulting from abortion. Therefore, this study examined post abortion modern contraceptive utilization among Ethiopian women as well as associated factors. The study was based on secondary data analysis of the Ethiopian Demographic and Health Survey 2016 data. A total weighted sample of 1236 reproductive age women was included. A multilevel mixed-effect binary logistic regression model was fitted to identify the significant associated factors of post abortion contraceptive use. Statistical significance was determined using Adjusted Odds Ratio (AOR) with 95% confidence interval. Overall prevalence of post abortion contraceptive use was observed to be 25.6% (95% CI: 23.24, 28.12). Women's age 15–24 (AOR = 2.34; 95% CI: 1.11, 4.93), and 25–34 (AOR = 1.94; 95% CI: 1.27, 2.98), married women (AOR = 2.6; 95% CI: 1.43, 4.96), women who had 1–4 (AOR = 4.13; 95% CI: 1.79, 9.57) and ≥ 5 number of children (AOR = 8.80; 95% CI: 3.30, 13.49), Being in metropolitan region (AOR = 9.14; 95% CI: 1.79, 12.48), women being in urban area (AOR = 1.85; 95% CI: 1.32, 2.24), and community media exposure (AOR = 1.75; 95% CI: 1.11, 3.56) were associated with post abortion modern contraceptive use. Post abortion modern contraceptive use in this study was low. Women age, current marital status, number of living children, residency, community media exposure, and region were significantly associated with post abortion modern contraceptive utilization. Therefore, it is better to provide ongoing health information about post-abortion family planning and its benefits, especially for people who live in rural and small peripheral regions, and public health policymakers should take both individual and community level factors into account when designing family planning programmes.

## Introduction

Post-abortion care involves various medical and related interventions to address women's health care needs after spontaneous and induced abortions^1,2^. Among its essential elements are emergency treatment for complications and contraceptives to prevent repeat abortions due to mistimed or unplanned pregnancies^2^. Post-abortion contraception refers to the use of family planning methods immediately following an abortion^1,2^.

Family planning services play a crucial role in reducing unmet family planning needs, increasing contraceptive usage, and preventing unintended pregnancy and unsafe abortion^3^. In addition it was investigated that there was appositive effects using ENG implants on improving quality of life, sexual function, and relief pelvic pain in women affected by endometriosis like ovarian cysts^4,5^. Positive changes in quality of life and sexual function were observed in postpartum women who used LNG-IUS^6^.

Globally, an estimated 121 million women experience unintended pregnancies each year, with 73.3 million women undergoing an abortion^7^. The majority of abortions occur in countries with low- and middle-incomes, and sub-Saharan Africa has a 27 abortion rate per 1000 women between 15 and 49 years^8^. Ethiopia is one of the countries with the highest number of abortions in sub-Saharan Africa, with 382,500 abortions annually^9^. Abortion has a direct contribution of 810 maternal deaths^8,10^. The number of deaths from unsafe abortion is also disproportionately high among women in sub-Saharan Africa, with 520 deaths per 100,000 women per year^7^.

To improve maternal health, the World Health Organization (WHO) recommends waiting six months to get pregnant after an abortion induced or spontaneous happened is important for maternal and child health^11^. By linking abortion care with family planning services, unmet need and subsequent unintended pregnancies are reduced^12^. According to studies conducted in Ethiopia, the prevalence of post-abortion family planning utilization varies greatly across the country, ranging from 45.8 to 91%^13–17^.

Several factors influence women's decisions to begin post-abortion family planning. According to studies conducted in Ethiopia, urban residence, higher maternal education level, occupation, undergoing spontaneous abortion, receiving post abortion family planning counseling, good knowledge of family planning, and availability of family planning are all significantly associated with post-abortion family planning uptake^18–22^.

In Ethiopia, a variety of strategies have been employed to increase the uptake of contraceptive methods over the last decade. Among the steps taken to increase contraceptive use was the implementation of health extension programs to change attitudes and improve awareness among the community^23,24^. Family planning as part of comprehensive package of post abortion care^25^ that is an important strategy to reduce maternal morbidity and mortality^26^ by breaking the cycle of repeated abortion and unintended pregnancy^27^. In spite of these efforts made at the national level, the proportion of women who use contraceptive methods remains low^28^.

We aimed therefore, to find out the prevalence and what factors are associated with the use of post-abortion modern contraception among Ethiopian women of reproductive age. It is hoped that the study's findings will assist policymakers in developing interventions that will help reduce maternal mortality and morbidity by increasing the use of post abortion modern contraceptives.

## Methods

### Study design, period, and setting

The study was a multilevel analysis of data from recent EDHS, which was conducted by the Central Statistical Agency (CSA) in collaboration with the Federal Ministry of Health (FMoH) and the Ethiopian Public Health Institute (EPHI), which was a national representative sample conducted from January 18 to June 27, 2016^29^.

There are nine regional states in Ethiopia (Tigray, Afar, Amhara, Oromia, Benishangul, Gambela, South Nation, Nationalities and Peoples’ Region (SNNPR), Harari, and Somali), and two administrative cities (Addis Ababa and Dire-Dawa), 611 Districts, and 15,000 Kebeles.

### Data sources

The data were gained from the official database of the EDHS program, https://www.measuredhs.com after authorization was granted via online request by explaining the purpose of our study. We extracted dependent and independent variables from the woman record (IR file). EDHS is a nationally representative household survey conducted by face-to-face interviews on a wide range of populations. Study participants were selected using a two-stage stratified sampling technique. Enumeration Areas (EAs) were randomly selected in the first stage, while households were selected in the second stage^30^. A total of weighted sample of 1236 post aborted reproductive age women were included.

### Variables and measurements

#### Dependent variable

The outcome variable was post-abortion modern contraceptive utilization. In the current study a woman was considered as modern contraceptive method utilizer if she had been using at least one of the modern contraceptives (female sterilization, male sterilization, IUCD, injectable, implants, pills, male condom, female condom, emergency contraception, and standard days method) during EDHS data collection period. Whereas a woman was considered to be non-utilizer of the modern contraceptive method if she had been using traditional methods like rhythm method, and withdrawal or if she had not been using any type of contraception during EDHS data collection period^31^.

#### Independent variables

##### Individual level variables

Age of respondents, educational status of respondents, current marital status, occupation of respondents, wealth index, media exposure, number of living children, visit health facility in the last 12 months, and religion were included.

##### Community level variables

Community level variables included residences and region was directly accessed from EDHS data sets. However, community level poverty, community level education, and community-level media exposure were constructed by aggregating individual-level characteristics at the cluster level^32^. They were categorized as high or low based on the distribution of the proportion values generated for each community after checking the distribution by using the histogram. The aggregate variable was not normally distributed and the median value was used as a cut-off point for the categorization^32,33^. Due to the non-normal distribution, we used the national median value to categorize these factors into high and low at community level. Poverty at the community level is calculated based on the proportion of households in the poorer and poorest quintiles. The proportion from a given community is grouped as low if it is less than 50% and as high if it is more than 50%. Community media exposure was categorized as low if the proportion of women exposed to media in the community was 0–26.8% and categorized as high if the proportion above 26.8%.

### Data analysis

For data analysis Stata version 16 software was used. To ensure the representativeness of the EDHS sample and obtain reliable estimations and standard errors, data were weighted (v005/1000000) throughout analysis.

Four models fitted: the null model with no explanatory variables, model I with individual factors, model II with community factors, and model III with both individual and community factors. To compare and assess the fitness of nested models, we used the intra class correlation coefficient (ICC), the median odds ratio (MOR), and deviation (−2LLR). Model III was the best-fitting model due to its low deviance. In multivariable analysis, variables with a p-value less than 0.2 in bivariable analysis were used. Finally, in the multivariable analysis, adjusted odds ratios with 95% confidence intervals and p-values less than 0.05 were used to identify factors of post abortion modern contraceptive utilization.

### Ethical approval and consent to participate

The study does not involve participants to provide information. Consent to participants is not applicable since the data is secondary and is available in the public domain. All the methods were conducted according to the Helsinki declarations. More details regarding EDHS data and ethical standards are available online at (http://www.dhsprogram.com). The study is not experimental study. Further explanation of how the DHS uses data and its ethical standards can be found at: http://goo.gl/ny8T6X.

## Results

### Individual level factors

Out of the total respondents, 759 (61.39%) women were not attended formal education, 686 (55.46%) of respondents were employed, and 694 (56.15%) of the respondents had no media exposure about family planning. Among the participants, 683 (55.25%) had 1–4 number of alive children. Among the respondents 1064 (86.32%) were married. With regard to their economic status, 451 (36.50%) women were from the poor wealth quintiles (Table 1).

**Table 1:** Individual characteristics of respondents in Ethiopia (n = 1236).

Variables	Categories	Frequency	Percentage (%)
Age of respondents	15–24	153	12.35
	25–34	460	37.25
	35–49	623	50.40
Educational status of respondents	No formal education	759	61.39
	Primary education	409	33.07
	Secondary and higher	69	5.54
Current marital status	Married	1067	86.32
	Unmarried	169	13.68
Occupation of respondents	Unemployed	550	44.54
	Employed	686	55.46
Wealth status	Poor	451	36.50
	Middle	243	19.64
	Rich	542	43.86
Media exposure	No	694	56.15
	Yes	542	43.85
Number of living children	None	104	8.45
	1–4	683	55.25
	≥ 5	449	36.30
Visit health facility in the last 12 months	No	603	48.79
	Yes	633	51.21
Religion	Orthodox	583	47.15
	Muslim	392	31.70
	Protestant	243	19.68
	Others	18	1.48

### Community level factors

Of the respondents 976 (79.01%) were rural dwellers. Among the respondents 1081 (87.50%) were from large central region. About 736 (59.63%) of the respondents were from communities with low proportion of poverty level. Above half (52.41%) of women had media exposure and 94.41% of the respondents were in communities with low community education level (Table 2).

**Table 2:** Community level characteristics of respondents in Ethiopia (n=1236).

Variables	Categories	Frequency	Percentage (%)
Residence	Rural	976	79.01
	Urban	260	21.02
Community level poverty	High	499	40.40
	Low	736	59.63
Community media exposure	Low	647	52.41
	High	589	47.64
Community level education	Low	1 167	94.41
	High	69	5.63
Region	Metropolitan	102	8.25
	Large central	1 081	87.50
	Small peripheral	52	4.24

### Post-abortion modern contraceptive utilization

**Figure 1:**
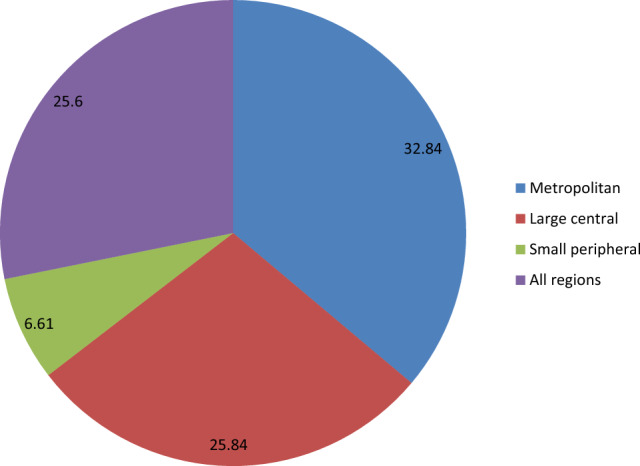
Prevalence of post abortion modern contraceptive utilization in Ethiopia.

Overall, the prevalence of post abortion modern contraceptive utilization in Ethiopia was 25.60% (95% CI: 23.24, 28.12), with metropolitan region recording the highest prevalence of 32.84% (Fig. 1).

### Factors associated with post-abortion modern contraceptive utilization

Study participants aged 15–24 years were 2.34 times more likely to use post abortion modern contraceptive (AOR = 2.34; 95% CI: 1.11, 4.93) as compared with those aged 35–49 years and the odds ratios of using post abortion modern contraceptive among aborted women in the age group of 25–34 were 1.94 times higher (AOR = 1.94; 95% CI: 1.27, 2.98) than those aged 35–49 years. Married participants were 2.6 times more likely to use post abortion modern contraceptive than unmarried participants with (AOR = 2.6; 95% CI: 1.43, 4.96).

Women who had 1–4 number of living children were 4.13 times modern contraceptive use (AOR = 4.13; 95% CI: 1.79, 9.57) than who had no children and women who had ≥ 5 living children were more likely to use modern contraceptive (AOR = 8.80 ; 95% CI: 3.30, 13.49) compared to women who had no living children.

Regarding community level factors, the odds of post abortion modern contraceptive utilization was high among women who had community media exposure (AOR = 1.75; 95% CI: 1.11, 3.56) compared with their counterparts. Women who were residing in urban area were 1.85 times contraceptive use than living in rural area (AOR = 1.85; 95% CI: 1.32, 2.24).

In addition, women in metropolitan region were 9.14 times more likely (AOR = 9.14; 95% CI: 1.79, 12.48) to use modern contraceptive compared to women in small peripheral region and women in large central region were 4.77 times more likely (AOR = 4.77; 95% CI: 1.18, 9.25) to use modern contraceptive compared to women in small peripheral region (Table 3).

**Table 3:** multivariable analyses for factors affecting modern contraceptive utilization **(**n = 1236).

Variables	Model 0	Model 1 AOR (95% CI)	Model 2 AOR (95%CI)	Model 3 AOR (95%CI)
Individual level variables				
Age				
15–24		2.11 (1.02, 4.38)		2.34 (1.11, 4.93)*
25–34		1.87 (1.23, 2.86)		1.94 (1.27, 2.98)*
35–49		1		1
Educational status				
No formal education		1		1
Primary education		1.45 (0.94, 2.29)		1.38 (0.87, 2.19)
Secondary and higher		0.85 (0.35, 2.05)		0.63 (0.23, 1.72)
Current marital status				
Married		2.50 (1.37, 4.56)		2.6 (1.43, 4.96)*
Not married		1		1
Occupation				
Employed		1.23 (0.83, 1.82)		1.10 (0.74, 1.65)
Not employed		1		1
Wealth status				
Poor		1		1
Middle		1.00 (0.57, 1.75)		0.85 (0.45, 1.59)
Rich		1.41 (0.84, 2.35)		1.09 (0.57, 2.08)
Media exposure				
No		1		1
Yes		1.25 (0.80, 1.93)		1.02 (0.60, 1.65)
Number of living children				
None		1		1
One to four		3.46 (1.52, 7.84)		4.13 (1.79, 9.57)*
≥ Five		6.31 (2.46, 16.23)		8.80 (3.30, 13.49)*
Visit health facility in the last 12 months				
No		1		1
Yes		0.98 (0.67, 1.43)		0.90 (0.6, 1.34)
Religion				
Orthodox		1		1
Muslim		0.97 (0.52, 1.82)		0.53 (0.28, 1.03)
Protestant		0.28 (0.16, 0.50)		1.45 (0.67, 3.12)
+ Others		0.05 (0.01, 1.30)		0.08 (0.00, 2.18)
Community level variables				
Residency				
Rural			1	1
Urban			0.65 (0.26, 1.57)	1.85 (1.32, 2.24)*
Community level media exposure				
Low			1	1
High			1.89 (1.04, 3.44)	1.75 (1.11, 3.56)*
Community level poverty				
High			1	1
Low			1.30 (0.71, 2.42)	1.32 (0.61, 2.86)
Community level education				
Low			1	1
High			0.88 (0.30, 2.53)	1.44 (0.43, 4.83)
Region				
Metropolitan			8.79 (1.88, 10.97)	9.14 (1.79, 12.48)*
Large central			7.52 (2.02, 11.10)	4.77 (1.18, 9.25)*
Small peripheral			1	1
Random effect results				
Variance (%)	4.37	2.78	2.37	2.11
ICC (%)	45.6	40.0	32.1	21.4
MOR	5.4	4.3	3.98	3.75
PCV	Ref	36.4	45.8	51.7
Deviance(−2LLR)	1458	1206	1074	1006

Model 2: adjusted for community-level characteristics.

Model 3: adjusted for both individual and community level characteristics.

+ Others = catholic, traditional and other EDHS category.

*AOR* adjusted odds ratio, *COR* crude odds ratio, *Null model* adjusted for individual-level characteristics.

*Statistically significant at p-value < 0.05.

## Discussion

The actual clinical implication of low post-abortion modern contraceptive utilization is considered one of the main and leading reasons of induced abortion, spontaneous abortion, or stillbirth because the majority of post-abortion women are almost immediately at risk for pregnancy. Therefore, aim of this study was to investigate the prevalence and predictors of post abortion modern contraceptive utilization among reproductive age women in Ethiopia. Only one forth 25.6% (95% CI: 23.24, 28.12) of participants were using post abortion modern contraceptive. This finding is lower than studies conducted in Ethiopia^14,17,19,34–37^, and other countries such as Mexico^38^, Nepal^39^, and Pakistan^40^. Socioeconomic status, the proportion of married women, research sites, periods, sample sizes, counseling, the accessibility of contraceptives, participant misconceptions about family planning methods, and social norms may all contribute to these disparities.

Being 15–24 and 25–34 years were 2.34 and 1.94 times more likely to utilize post abortion contraceptive than their counterparts respectively. This finding is supported by the study conducted in Gambella^41^ and Bahir Dar^35^. This implies that the government should prioritize meeting the family planning needs of those groups in order to avoid early pregnancy and childbirth-related complications^41,42^. This study pointed out that married women were 2.6 times more likely to utilize post abortion contraceptives than their counterparts. This is supported by studies conducted in a different part of Ethiopia^42–44^. One possible explanation is that married women live with their husbands, making them more prone to sexual exposure.

The use of post abortion modern contraceptive methods was independently associated with the number of children. In comparison to a woman who had no children, women who had 1–4 children and women who had five and above children were four and nearly nine times more likely respectively, to have used post abortion modern contraception. Additionally, prior studies have shown that using modern contraceptives is more likely when there is adequate number of living children^29,45,46^. This could be because women without children may require children in order to have the optimal number of children^47^.

In regard to community level factors, urban women of reproductive age were about1.85 times more likely than rural women to use post abortion contraceptives. This finding is in line with secondary data analysis of Indian, Afghan, Nigerian, and Bangladeshi Demographic and Health Surveys, which showed that urban resident women were more likely than rural resident women to use contemporary contraceptives^48–51^. This could be caused by a variety of factors. Urban women tend to be more educated, earn more money, have better access to health facilities, and have better media access than rural women, all of which lead to higher modern contraceptive utilization rates. A number of rural women also depend on children to assist them in field work, which negatively impacts the use of modern contraceptives among them^31,52–56^.

Women who were exposed to the community media had a higher likelihood of using modern methods of contraception than women who were not. The finding is in line with the results^29,48,57–59^. This is due to the possibility that exposure to mass media could play a significant role in raising awareness and inspiring women to take contemporary contraceptive. In addition woman living in metropolitan region and large central of Ethiopia were 9 and 4.77 times more likely to use post abortion modern contraceptive compared to women in small peripheral region. There may be differences in access to health information and different availability of maternal health services, such as family planning^60^.

This study used most recent nationally representative data, which were collected with validated and standardized data collection tools. This also employed multilevel analysis (advanced model) that accounts the correlated nature of EDHS data in the determination of the estimate. Despite the above advantages, the cross sectional nature of the study does not show the cause and effect relationship between the outcome and the independent factors. Moreover, due to EDHS were secondary data, essential factors like attitude and knowledge about contraceptives, partner’s perspectives on contraceptives, and fear of side effects were not available in the EDHS.

## Conclusion

Post-abortion modern contraceptives utilization in Ethiopia was low. Women age, current marital status, number of living children, residency, community media exposure, and region were significantly associated with post abortion modern contraceptive utilization. Therefore, it is better to provide ongoing health information about post-abortion family planning and its benefits, especially for people who live in rural and small peripheral regions, and public health policymakers should take both individual and community level factors into account when designing family planning programmes.

## Data Availability

Data for this study were sourced from Ethiopian Demographic and Health surveys (EDHS), which is freely available online at (https://dhsprogram.com).

## References

[1] WH Organization. *Handbook on Health Inequality Monitoring: With a Special Focus on Low-and Middle-Income Countries*. (World Health Organization, 2013).

[2] WH Organization. *WHO Recommendations for Augmentation of Labour*. (World Health Organization, 2014). 25506951

[3] Huber D, Curtis C, Irani L, Pappa S, Arrington L (2016). Postabortion care: 20 years of strong evidence on emergency treatment, family planning, and other programming components. Glob. Health Sci. Pract..

[4] Yisa SB, Okenwa AA, Husemeyer RP (2005). Treatment of pelvic endometriosis with etonogestrel subdermal implant (Implanon^®^). BMJ Sex. Reprod. Health..

[5] Walch K, Unfried G, Huber J, Kurz C, van Trotsenburg M, Pernicka E (2009). Implanon^®^ versus medroxyprogesterone acetate: Effects on pain scores in patients with symptomatic endometriosis—A pilot study. Contraception.

[6] Caruso S, Cianci S, Vitale SG, Fava V, Cutello S, Cianci A (2018). Sexual function and quality of life of women adopting the levonorgestrel-releasing intrauterine system (LNG-IUS 1.35 mg) after abortion for unintended pregnancy. Eur. J. Contracept. Reprod. Health Care..

[7] Kumar M, Daly M, De Plecker E, Jamet C, McRae M, Markham A (2020). Now is the time: A call for increased access to contraception and safe abortion care during the COVID-19 pandemic. BMJ Glob. Health.

[8] Bearak J, Popinchalk A, Ganatra B, Moller A-B, Tunçalp Ö, Beavin C (2020). Unintended pregnancy and abortion by income, region, and the legal status of abortion: Estimates from a comprehensive model for 1990–2019. Lancet Glob. Health.

[9] Assefa EM (2019). Knowledge, attitude and practice (KAP) of health providers towards safe abortion provision in Addis Ababa health centers. BMC Womens Health.

[10] Rahman MA, Halder HR, Islam SMS (2021). Effects of COVID-19 on maternal institutional delivery: Fear of a rise in maternal mortality. J. Glob. Health..

[11] WH Organization. *International Travel and Health: Situation as on 1 January 2007* (World Health Organization, 2007).

[12] Ceylan A, Ertem M, Saka G, Akdeniz N (2009). Post abortion family planning counseling as a tool to increase contraception use. BMC Public Health.

[13] Abate E, Smith YR, Kindie W, Girma A, Girma Y (2020). Prevalence and determinants of post-abortion family planning utilization in a tertiary Hospital of Northwest Ethiopia: A cross sectional study. Contracept. Reprod. Med..

[14] Asrat M, Bekele D, Rominski SD (2018). Post-abortion contraceptive acceptance and choice among women receiving abortion care at Saint Paul's Hospital, Addis Ababa, Ethiopia: A cross-sectional study. Lancet Glob. Health.

[15] Baynes C, Yegon E, Lusiola G, Achola J, Kahando R (2021). Post-abortion fertility desires, contraceptive uptake and unmet need for family planning: Voices of post-abortion care clients in Tanzania. J. Biosoc. Sci..

[16] Muche A, Bewket B, Ayalew E, Demeke E (2019). Utilization of post abortal contraceptive use and associated factors among women who came for abortion service at Debre Berhan Hospital, Debre Berhan, Ethiopia March 2019: institution based cross sectional study. Clin. J. Obstet. Gynecol..

[17] Muchie A, Getahun FA, Bekele YA, Samual T, Shibabaw T (2021). Magnitudes of post-abortion family planning utilization and associated factors among women who seek abortion service in Bahir Dar Town health facilities, Northwest Ethiopia, facility-based cross-sectional study. PLoS ONE.

[18] Kokeb L, Admassu E, Kassa H, Seyoum T (2015). Utilization of post abortion contraceptive and associated factors among women who came for abortion service: a hospital based cross sectional study. J. Fam. Med. Dis. Prev..

[19] Prata N, Bell S, Holston M, Gerdts C, Melkamu Y (2011). Factors associated with choice of post-abortion contraception in Addis Ababa, Ethiopia. Afr. J. Reprod. Health.

[20] Amentie M, Abera M, Abdulahi M (2015). Utilization of family planning services and influencing factors among women of child bearing age in Assosa district, Benishangul Gumuz regional state, West Ethiopia. Sci. J. Clin. Med..

[21] Zemene A, Feleke A, Alemu A, Yitayih G, Fantahun A (2014). Factors influencing utilization of post abortion care in selected Governmental Health Institutions, Addis Ababa. Ethiopia. Fam Med Med Sci Res..

[22] Dejenie Seyoum, A.G., & Gizaw, Z. *Assessment of Post Abortion Contraceptive Intention and Associated Factors Among Abortion Clients in Gondar Town, North West Ethiopia, 2013* (2014).

[23] Sedlander E, Bingenheimer JB, Edberg MC, Rimal RN, Shaikh H, Munar W (2018). Understanding modern contraception uptake in one Ethiopian community: A case study. Reprod. Health.

[24] Kahsay ZH, Hiluf MK, Shamie R, Tadesse Y, Bazzano AN (2019). Pregnant Women’s intentions to deliver at a health Facility in the Pastoralist Communities of Afar, Ethiopia: An application of the health belief model. Int. J. Environ. Res. Public Health.

[25] Tripney J, Kwan I, Bird KS (2013). Postabortion family planning counseling and services for women in low-income countries: A systematic review. Contraception.

[26] Opoku B (2012). Contraceptive preferences of post-abortion patients in Ghana. J. Women’s Health Care..

[27] Seid A, Gebremariam A, Abera M (2012). Integration of family planning services within post abortion care at health facilities in dessie—North East Ethiopia. Sci. Technol. Arts Res. J..

[28] Fantay Gebru K, Mekonnen Haileselassie W, Haftom Temesgen A, Oumer Seid A, Afework MB (2019). Determinants of stunting among under-five children in Ethiopia: a multilevel mixed-effects analysis of 2016 Ethiopian demographic and health survey data. BMC Pediatr..

[29] Abate MG, Tareke AA (2019). Individual and community level associates of contraceptive use in Ethiopia: A multilevel mixed effects analysis. Arch. Public Health.

[30] Corsi DJ, Neuman M, Finlay JE, Subramanian S (2012). Demographic and health surveys: A profile. Int. J. Epidemiol..

[31] Gebre MN, Edossa ZK (2020). Modern contraceptive utilization and associated factors among reproductive-age women in Ethiopia: Evidence from 2016 Ethiopia demographic and health survey. BMC Womens Health.

[32] Liyew AM, Teshale AB (2020). Individual and community level factors associated with anemia among lactating mothers in Ethiopia using data from Ethiopian demographic and health survey, 2016; A multilevel analysis. BMC Public Health.

[33] Getaneh T, Negesse A, Dessie G, Desta M, Moltot T (2020). Predictors of unmet need for family planning in Ethiopia 2019: A systematic review and meta analysis. Arch. Public Health..

[34] Abebe AM, Wudu Kassaw M, Estifanos SN (2019). Postabortion contraception acceptance and associated factors in Dessie Health Center and Marie Stopes International Clinics, South Wollo Northeast, Amhara region, 2017. Int. J. Reprod. Med..

[35] Mekuria A, Gutema H, Wondiye H, Abera M (2019). Postabortion contraceptive use in Bahir Dar, Ethiopia: A cross sectional study. Contracept. Reprod. Med..

[36] Tesfaye G, Oljira L (2013). Post abortion care quality status in health facilities of Guraghe zone, Ethiopia. Reprod. Health.

[37] Chukwumalu K, Gallagher MC, Baunach S, Cannon A (2017). Uptake of postabortion care services and acceptance of postabortion contraception in Puntland, Somalia. Reprod. Health Matters.

[38] Becker D, Olavarrieta CD, Garcia SG, Harper CC (2013). Women's reports on postabortion family-planning services provided by the public-sector legal abortion program in Mexico City. Int. J. Gynecol. Obstet..

[39] Khanal V, Joshi C, Neupane D, Karkee R (2011). Practices and perceptions on contraception acceptance among clients availing safe abortion services in Nepal. Kathmandu Univ. Med. J..

[40] Azmat SK, Hameed W, Ishaque M, Mustafa G, Ahmed A (2012). Post-abortion care family planning use in Pakistan. Pak. J. Public Health..

[41] Abamecha, A., Shiferaw, A. & Kassaye, A. *Assessment of Post Abortion Contraceptive Intention and Associated Factors Among Abortion Clients in Gambella Health Facilities, Gambella Town, South West Ethiopia. Gambella Town, South West Ethiopia* (2016).

[42] Motuma VS, Yadeta TA, Alemu A, Yuya M, Eshetu B, Balis B (2022). Postabortion family planning and associated factors among women attending abortion service in Dire Dawa Town Health Facilities, Eastern Ethiopia. Front. Reprod. Health..

[43] Bizuneh AD, Azeze GG (2021). Post-abortion family planning use, method preference, and its determinant factors in Eastern Africa: A systematic review and meta-analysis. Syst. Rev..

[44] Moges Y, Hailu T, Dimtsu B, Yohannes Z, Kelkay B (2018). Factors associated with uptake of post-abortion family planning in Shire town, Tigray, Ethiopia. BMC Res. Notes.

[45] Idris H (2019). Factors affecting the use of contraceptive in Indonesia: Analysis from the National Socioeconomic Survey (Susenas). KEMAS J. Kesehatan Masyarakat..

[46] Devita VD, Rosliza A, Suriani I (2018). Prevalence of modern contraceptive use among reproductive women with hypertension and diabetes in a government hospital in BATAM, Indonesia and its socio-demographic determinants. Int. J. Public Health Clin. Sci..

[47] Naigino R, Makumbi F, Mukose A, Buregyeya E, Arinaitwe J, Musinguzi J (2017). HIV status disclosure and associated outcomes among pregnant women enrolled in antiretroviral therapy in Uganda: A mixed methods study. Reprod. Health.

[48] Fenta SM, Gebremichael SG (2021). Predictors of modern contraceptive usage among sexually active rural women in Ethiopia: A multi-level analysis. Arch. Public Health..

[49] Ezeh OK, Agho KE, Dibley MJ, Hall J, Page AN (2014). The impact of water and sanitation on childhood mortality in Nigeria: Evidence from demographic and health surveys, 2003–2013. Int. J. Environ. Res. Public Health.

[50] Haq I, Sakib S, Talukder A (2017). Sociodemographic factors on contraceptive use among ever-married women of reproductive age: Evidence from three demographic and health surveys in Bangladesh. Med. Sci..

[51] Dey AK (2019). Socio-demographic determinants and modern family planning usage pattern—An analysis of National Family Health Survey-IV data. Int. J. Commun. Med. Public Health.

[52] Tekelab T, Melka AS, Wirtu D (2015). Predictors of modern contraceptive methods use among married women of reproductive age groups in Western Ethiopia: A community based cross-sectional study. BMC Womens Health.

[53] Johnson OE (2017). Determinants of modern contraceptive uptake among Nigerian women: Evidence from the national demographic and health survey. Afr. J. Reprod. Health.

[54] Osmani AK, Reyer JA, Osmani AR, Hamajima N (2015). Factors influencing contraceptive use among women in Afghanistan: secondary analysis of Afghanistan Health Survey 2012. Nagoya J. Med. Sci..

[55] Adebowale AS, Gbadebo B, Afolabi FR (2016). Wealth index, empowerment and modern contraceptive use among married women in Nigeria: Are they interrelated?. J. Public Health.

[56] Adebowale SA, Adedini SA, Ibisomi LD, Palamuleni ME (2014). Differential effect of wealth quintile on modern contraceptive use and fertility: Evidence from Malawian women. BMC Womens Health.

[57] Agbadi, P., Tagoe, E., Akosua, A.F. & Owusu, S. *A Multilevel Analysis of Predictors of Modern Contraceptive Use Among Reproductive Age Women in Sierra Leone: Insight from Demographic and Health Surveys*. (Center for Open Science, 2019).

[58] Rutaremwa G, Kabagenyi A, Wandera SO, Jhamba T, Akiror E, Nviiri HL (2015). Predictors of modern contraceptive use during the postpartum period among women in Uganda: A population-based cross sectional study. BMC Public Health.

[59] Nsanya MK, Atchison CJ, Bottomley C, Doyle AM, Kapiga SH (2019). Modern contraceptive use among sexually active women aged 15–19 years in North-Western Tanzania: Results from the Adolescent 360 (A360) baseline survey. BMJ Open.

[60] Ababa A. Ethiopia. *Abstract*. https://wfpha.confex.com/wfpha/2012/webprogram.Paper10587html[GoogleScholar] (2013).

